# The role of impaired bone marrow Tregs in hematopoietic stem cell depletion for pediatric aplastic anemia probably involves immune privilege

**DOI:** 10.1007/s44313-026-00141-6

**Published:** 2026-05-26

**Authors:** Can Huang, Shanshan Li, Jingwei Yang, Yangyang Jiao, Ting Zhang, Hui Jiang, Fanyi Zeng, Shayi Jiang

**Affiliations:** 1 Department of Hematology and Oncology, School of Medicine, Shanghai Children’s Hospital, Shanghai Jiao Tong University, Shanghai, China; 2 Institute of Pediatric Infection, Immunity and Critical Care Medicine, School of Medicine, Shanghai Children’s Hospital, Shanghai Jiao Tong University, Shanghai, China; 3 Shanghai Institute of Medical Genetics, Shanghai Children’s Hospital, & Department of Histo-Embryology, Genetics and Developmental Biology, School of Medicine, Shanghai Jiao Tong University, Shanghai, China; 4 NHC Key Laboratory of Medical Embryogenesis and Developmental Molecular Biology & Shanghai Key Laboratory of Embryo and Reproduction Engineering, Shanghai, China

**Keywords:** Aplastic anemia, Immune privilege, Hematopoietic stem cells, Immune microenvironment, Regulatory T cells

## Abstract

**Purpose:**

An immune-mediated pathogenesis has been postulated for aplastic anemia (AA), and impaired Tregs and other immune abnormalities have been identified in patients with AA. However, the process by which abnormal immunity specifically damages hematopoietic stem cells (HSCs) remains unclear. We conducted primary clinical studies to investigate whether the depletion of HSCs in AA could be attributed to the collapse of immune privilege (IP) afforded by regulatory T cells (Tregs).

**Methods:**

The distribution of Tregs in the bone marrow of children with AA, myelodysplastic syndrome (MDS), and control participants was separately detected by immunohistochemistry. T-helper 1 (Th1), T-helper 2 (Th2), and T-helper 17 (Th17) cells, cytokines, and HSCs in the bone marrow of the AA and control groups were examined using flow cytometry.

**Results:**

Patients with AA showed significantly lower FoxP3 + /CD4 + ratios (AA *vs*. normal: 18.09% ± 5.38% *vs*. 21.72% ± 4.21%, *P* = 0.014; AA *vs.* MDS: 18.09% ± 5.38% *vs*. 22.63% ± 5.98%, *P* = 0.030) and reduced Treg counts (AA *vs*. normal: 4.07 ± 1.41 *vs*. 5.25 ± 1.86 cells/HP, *P* = 0.014; AA *vs.* MDS: 4.07 ± 1.41 *vs*. 5.30 ± 1.49 cells/HP, *P* = 0.024) near the endosteum. The bone marrow in patients with AA exhibited Th1 dominance (AA *vs*. normal: 39.80% ± 5.20% *vs*. 22.1% ± 2.9% in controls, *P* < 0.001) and Th2 reduction (AA *vs*. normal: 59.20% ± 5.30% *vs*. 77.20% ± 2.60%, *P* < 0.001), indicating IP dysfunction. Significant elevation of tumor necrosis factor (TNF)-α, interferon (IFN)-γ, and interleukin (IL)-17 levels was observed in the bone marrow of patients with AA. Long-term (LT)-HSCs were severely depleted in patients with AA (AA *vs*. normal: 0.13% *vs*. 1.22%, *P* = 0.035), with moderate reduction in short-term (ST)-HSCs.

**Conclusions:**

Patients with AA showed endosteal Treg reduction, Th1 dominance, elevated proinflammatory cytokine levels, and severe LT-HSC depletion, linking bone marrow IP dysfunction to HSC damage. These findings provide a mechanistic explanation for the specific loss of HSCs in AA and highlight the IP niche as a therapeutic target.

**Supplementary Information:**

The online version contains supplementary material available at https://doi.org/10.1007/s44313-026-00141-6.

## Introduction

Acquired aplastic anemia (AA) is characterized by pancytopenia caused by bone marrow failure without dysplasia or fibrosis. Both clinical and laboratory evidence support the idea that the pathogenesis of bone marrow failure is injury to hematopoietic stem cells (HSCs) due to abnormal immunity [1, 2]. Overall, research on HSC depletion in AA has focused on exploring the mechanisms by which abnormal immunity attacks HSCs. However, to date, no specific antibodies or immune cells targeting HSCs have been identified. Therefore, the “immune-mediated pathogenesis” of AA has not yet clarified how HSCs, rather than other cells, are damaged by immune disorders in the bone marrow.

An in-depth study of the bone marrow microenvironment revealed sites with immune privilege (IP) in the bone marrow specific to HSCs [3]. These IP sites consisted of CD4^+^, CD25^+^,and FoxP3^+^ regulatory T cells (Tregs) that surround HSCs near the surface of bone trabeculae in the bone marrow, providing an immune-tolerant microenvironment for HSCs to protect them from infection, radiation, and other stress conditions. Tregs have been found to decrease in the peripheral blood and bone marrow of individuals with AA [4], indicating the need to shift the research on depletion mechanisms of HSCs in AA from “immune attack” to “loss of IP protection.” IP sites also exist in other tissues, and their abnormal pathogenicity has been a concern in various diseases [5]. For example, IP sites in hair follicles provide protection to epithelial hair follicle stem cells, and functional impairment of these IP sites leads to the destruction of epithelial hair follicle stem cells, which is the principal pathological mechanism of persistent alopecia in alopecia areata [6]. However, the role of bone marrow IP sites in the pathogenesis of AA has not been reported. The immune tolerance status of IP sites is usually determined by detecting local FoxP3^+^/CD4^+^ cells in tissues using an immunohistochemical method [7] and evaluating the T-helper 1 (Th1)/T-helper 2 (Th2) cell ratio. The levels of certain cytokines also indirectly reflect the function of IP [8, 9]. This study aimed to investigate the immune tolerance status of the bone marrow microenvironment provided by IP sites to HSCs in children with AA and to speculate its possible involvement in HSC exhaustion.

## Methods

### Patients

Thirty patients with AA and ten patients with myelodysplastic syndrome (MDS) were recruited from Shanghai Children's Hospital. Twenty children with solid tumors without bone marrow infiltration or hematological complications served as normal controls. Bone marrow tissue specimens for immunohistochemical tests were obtained from all patients, including those with AA and MDS, and the controls. Bone marrow fluid from the first suction (minimizing the dilution of bone marrow fluid by blood as much as possible) and peripheral blood were collected from patients with AA and the normal controls for flow cytometry detection.

### Immunohistochemical staining for FoxP3, CD4, and CD3

Immunohistochemical staining for FoxP3, CD4, and CD3 was performed separately using the Ventana Benchmark GX Immuno-AutoStainer (Roche) according to the manufacturer’s instructions. Primary anti-human rabbit monoclonal antibodies, including anti-FoxP3 (Abcam), anti-CD4 (Abcam), and anti-CD3 (Abcam), were detected using an indirect biotin streptavidin system (HRP/DAB Detection ICH kits, Abcam). FoxP3^+^ cells presented a brown chromogen in the nucleus, whereas CD4^+^ and CD3^+^ cells showed cytoplasmic brown granules.

All the slides were independently reviewed by two skilled pathologists. The positive cells were counted in five visual fields near the trabecular bone endosteum. The distance from the positive cells to the endosteum was measured. ​​ The mean ratio of FoxP3^+^/CD4^+^ cells in each case represented the Treg-mediated local immune tolerance status in the bone marrow.

### Flow cytometry analysis of Th1, Th2, and Th17 cells, HSCs, and cytokines

Th1, Th2, and T-helper 17 (Th17) cells were detected in the bone marrow of children with AA and controls using flow cytometry. These analyses were performed in accordance with the protocol of the ENTIRE HIP-C project [10]. For HSCs, long-term HSCs (LT-HSCs) and short-term HSCs (ST-HSCs) were sorted as previously described [11]. The anti-human antibodies (BD Pharmingen) included V500-CD3, APC-H7-CD4, PerCP-CD8, FITC-CXCR3, V450-CCR6, PE-CCR7, FITC-CD34, PE-Cy7-CD38, PE-Cy7-CD45RA, PE-CD90, Alexa Fluor 647-CD49f, PerCP-Cy5.5-CD3, PerCP-Cy5.5-CD56, PerCP-Cy5.5-CD11, and PerCP-Cy5.5-CD19. Th1, Th2, and Th17 cells were identified by lineages CD3^+^CD4^+^ CD45RA^−^CCR7^−^CXCR3^+^CCR6^−^, CD3^+^CD4^+^ CD45RA^−^CCR7^−^CXCR3^−^CCR6^−^, and CD3^+^CD4^+^CD45RA^−^CCR7^−^CXCR3^−^ CCR6^+^, respectively. Lineage^−^ (Lin^−^)​​ refers to cell surface antigens of CD3^−^CD19^−^CD11^−^CD56^−^. LT-HSCs presented as lin^−^CD34^+^CD38^−^CD45RA^−^CD90^+^CD49f^+^, whereas ST-HSCs presented as lin^−^CD34^+^CD38^−^CD45RA^−^CD90^−^CD49f^−^. Monocytes were collected from the bone marrow aspirates or peripheral blood using a Ficoll density gradient. The cells were then analyzed using flow cytometry. The analysis strategy is shown in Supplementary Figure S1 and S2. Data analysis was performed using FlowJo software.

Cytokines in the bone marrow and peripheral blood were detected using multiplex bead-based flow cytometric immunoassays. Multiplex assay kits for interleukin (IL)−5, tumor necrosis factor (TNF)-α, IL-2, IL-6, IL-1β, IL-10, interferon (IFN)-γ, IL-8, IL-17, and IL-12 were purchased from RAISECARE in China. These cytokines were tested in plasma samples obtained by centrifugation of heparinized bone marrow or peripheral blood from children with AA and control children.

### Statistical analysis

All graphs and statistical analyses were performed using GraphPad Prism version 9.4.1. Continuous variables were presented as mean ± standard deviation (SD). As the data were not normally distributed, nonparametric Mann–Whitney and Kruskal–Wallis tests were used for data comparison. *P* < 0.05 was considered as statistically significant.

### Ethical approval

This study was approved by the Children’s Hospital of Shanghai Local Research Ethics Committee, and written informed consent was obtained from the patients’ parents.

## Results

### The IP sites of patients with AA showed fewer Tregs

**Fig. 1:**
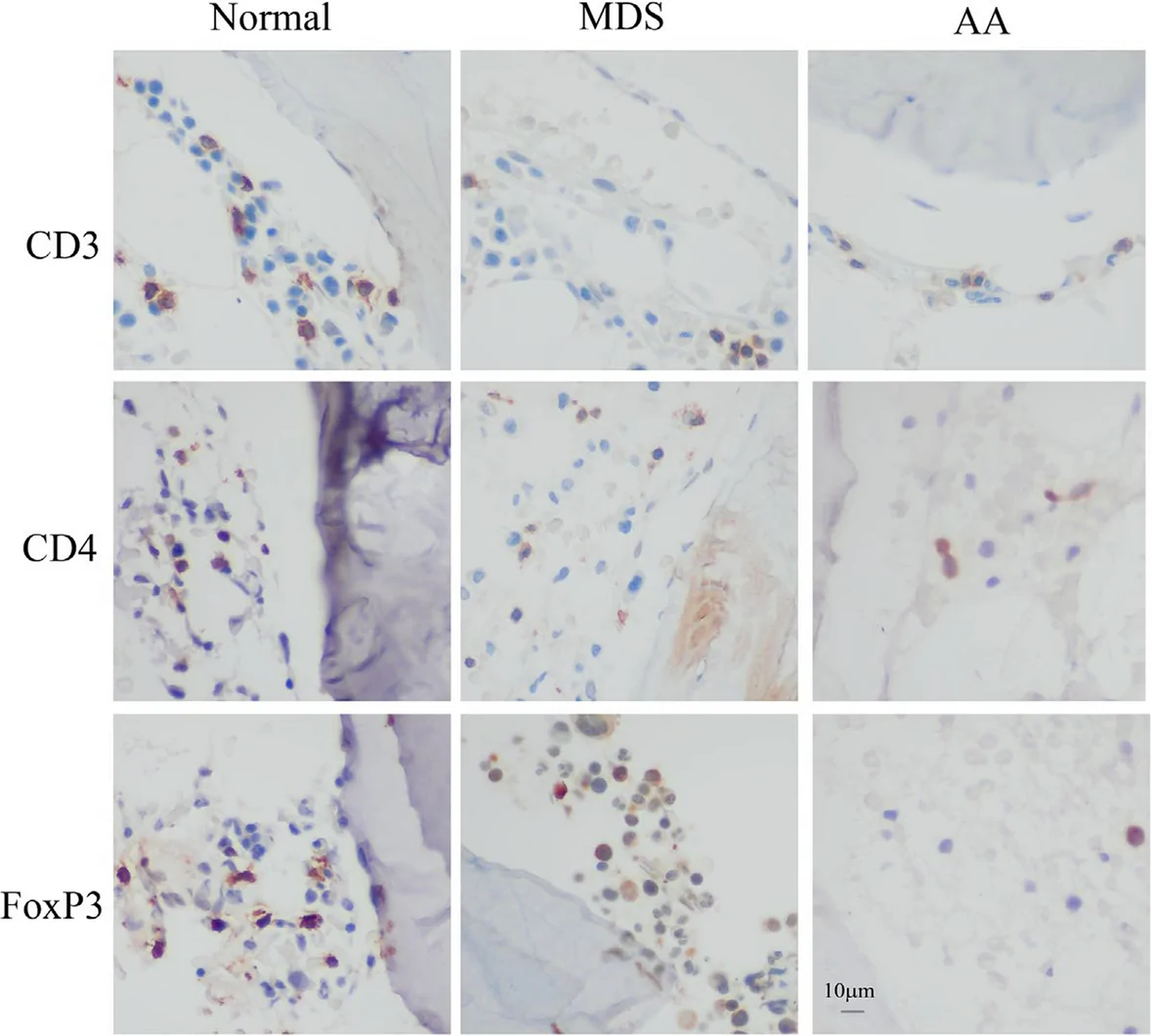
CD3^+^, CD4^+^, and FoxP3^+^ cells near the endosteum detected by immunohistochemistry. CD3^+^, CD4^+^, and FoxP3^+^ cells all decreased in patients with AA

HSCs were located near the endosteum, which has been identified as an IP site, forming the osteoblast niche [12], where Tregs accumulated and protected HSCs via IP mechanisms. Therefore, the local FoxP3^+^/CD4^+^ cells near the endosteum should indicate the immune tolerance status provided by IP to HSCs. We observed FoxP3^+^, CD4^+^, and CD3^+^ cells near the endosteum (Fig. 1).

**Fig. 2:**
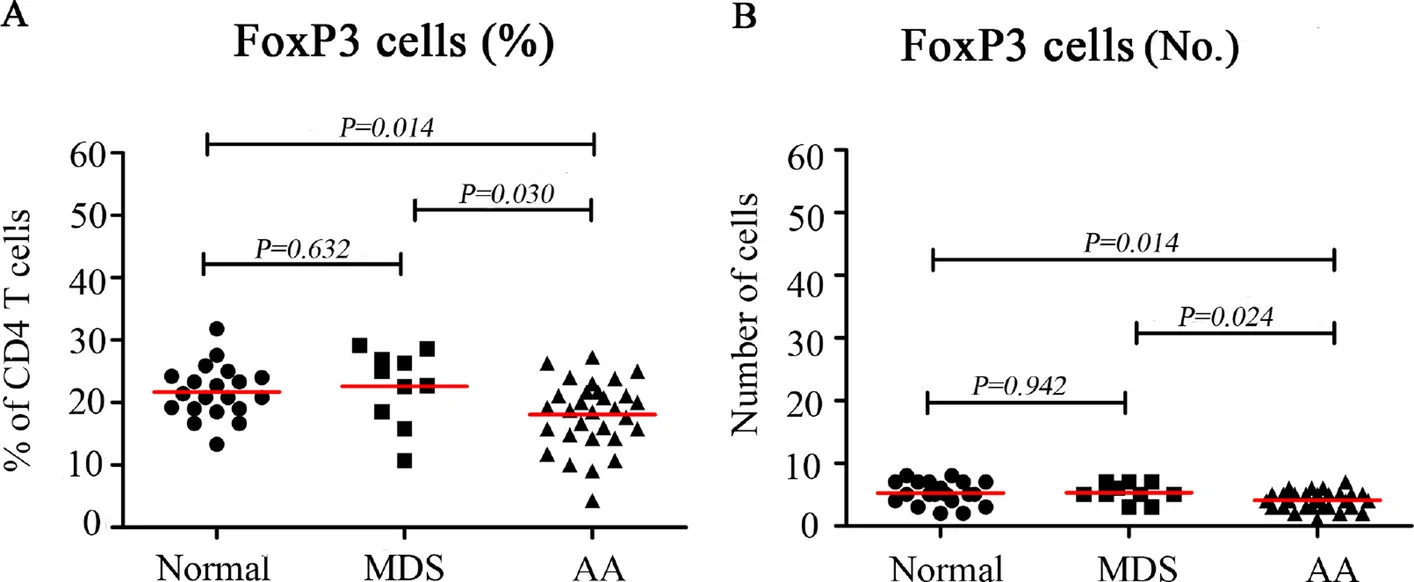
FoxP3^+^/CD4^+^ cells (**A**) and absolute number of FoxP3^+^ cells (**B**) in normal controls and patients with MDS and AA. Both FoxP3^+^/CD4^+^ and FoxP3^+^ cell counts in patients with AA were lower than those in normal controls and patients with MDS

In accordance with studies localizing HSCs and Tregs [12, 13], we enumerated positive cells (Tregs) within 45 μm from the endosteum surface. FoxP3^+^/CD4^+^ cells were significantly lower in patients with AA than in normal controls (18.09% ± 5.38% *vs*. 21.72% ± 4.21%, *P* = 0.014) and patients with MDS (18.09% ± 5.38% *vs*. 22.63% ± 5.98%, *P* = 0.030) (Fig. 2A). Considering the low number of Tregs in the bone marrow, we used the absolute count of Tregs to represent the immune tolerance microenvironment, and the average number of Tregs in AA was also significantly lower than that in the normal control (4.07 ± 1.41 *vs*. 5.25 ± 1.86 cells/HP, *P* = 0.014) and MDS (4.07 ± 1.41 *vs*. 5.30 ± 1.49 cells/HP, *P* = 0.024) (Fig. 2B), irrespective of the absolute counts of FoxP3^+^/CD4^+^ or FoxP3^+^ cells.

### Th1/Th2 imbalance in AA

**Fig. 3:**
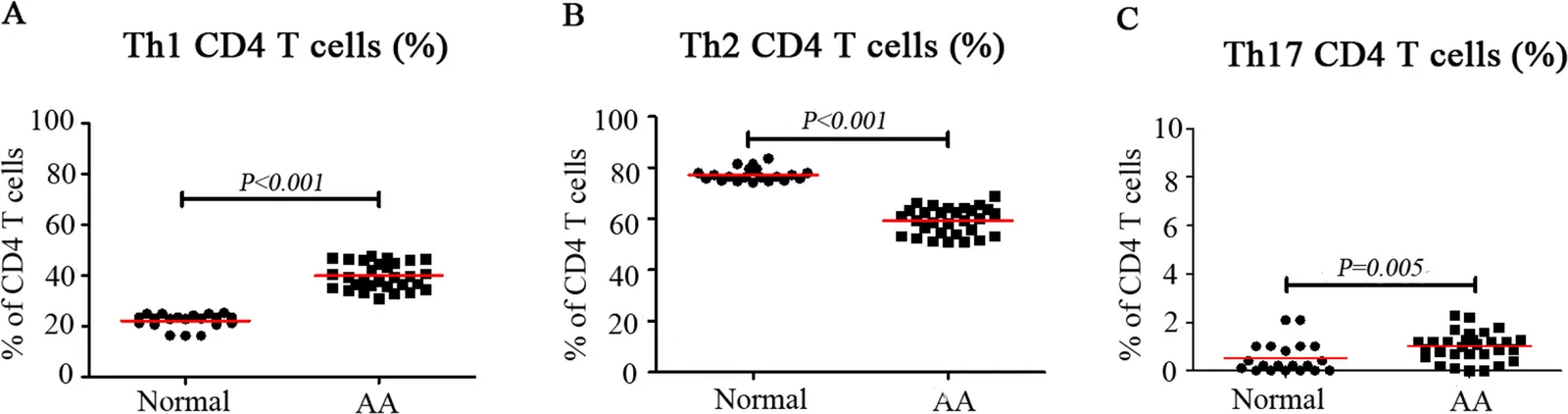
Proportion of Th1, Th2, and Th17 cells in the bone marrow. The percentages of Th1 (**A**), Th2 (**B**), and Th17 (**C**) cells in normal controls and patients with AA measured by flow cytometry. In comparison with the normal control group, the AA group showed significantly higher percentages of Th1 and Th17 cells

The normal IP effect also involves homeostasis between Th1 and Th2 cells, with a predominance of Th2 cells and relatively low activity of Th1 and Th17 cells. Therefore, the Th1/Th2 ratio indirectly reflects IP function [8, 14, 15]. Th17 cells are also considered to be similar to Th1 cells in the regulation of immune tolerance. In comparison with the normal controls, the AA group showed a significantly higher proportion of Th1 cells and a lower proportion of Th2 cells in the bone marrow (for Th1 cells, 39.80% ± 5.20% *vs*. 22.1% ± 2.9%, *P* < 0.001; for Th2 cells, 59.20% ± 5.30% *vs*. 77.20% ± 2.60%, *P* < 0.001), and a shift in the Th1/Th2 ratio. Meanwhile, the number of Th17 cells in the AA group was higher than that in the control group (1.00% ± 0.60% *vs*. 0.50% ± 0.70%, *P* = 0.005) (Fig. 3). The predominance of Th1 cells in the bone marrow also supported the weakening of the local immune tolerance environment.

### Cytokines in the bone marrow of patients with AA

Significant increments in the levels of TNF-α, IFN-γ, and IL-17 were observed in the bone marrow of patients with AA in comparison with those in the normal controls (TNF-α: 11.20 ± 35.20 *vs*. 2.80 ± 0.70 pg/mL, *P* < 0.001; IFN-γ: 35.00 ± 7.80 *vs*. 13.00 ± 5.80 pg/mL, *P* < 0.001; and IL-17: 28.90 ± 11.20 *vs* 2.60 ± 0.30 pg/mL, *P* < 0.001, respectively; Table 1). The levels of TNF-α, IFN-γ, and IL-17 in the peripheral blood of patients in the AA group were comparable or lower than those in the control group, but they showed no statistically significant differences.

**Table 1:** Flow cytometry analysis of the expression levels of cytokines in bone marrow aspirates and peripheral blood

	Bone marrow			Peripheral Blood		
Cytokine	Control	AA	*P* value	Control	AA	*P* value
IL-5	3.31 ± 0.64	3.61 ± 0.93	*P* = 0.318	2.95 ± 0.70	2.95 ± 0.62	*P* = 0.604
TNF-α	2.80 ± 0.70	11.20 ± 35.20	*P* < 0.001	3.49 ± 0.65	2.71 ± 0.22	*P* = 0.406
IL-2	1.28 ± 0.55	0.97 ± 0.50	*P* = 0.116	1.52 ± 0.46	1.79 ± 0.42	*P* = 0.182
IL-6	5.56 ± 1.39	6.31 ± 1.53	*P* = 0.185	4.54 ± 1.88	4.86 ± 1.06	*P* = 0.664
IL-1β	0.40 ± 0.45	0.78 ± 0.55	*P* = 0.109	0.87 ± 0.48	0.86 ± 0.46	*P* = 0.964
IL-10	3.58 ± 1.07	3.38 ± 1.67	*P* = 0.796	4.31 ± 1.66	3.03 ± 1.94	*P* = 0.132
IFN-γ	13.00 ± 5.80	35.00 ± 7.80	*P* < 0.001	4.26 ± 1.03	4.19 ± 0.79	*P* = 0.791
IL-8	1.40 ± 0.49	1.78 ± 0.53	*P* = 0.087	1.59 ± 0.43	1.25 ± 0.49	*P* = 0.136
IL-17	2.60 ± 0.30	28.90 ± 11.20	*P* < 0.001	3.19 ± 0.39	3.07 ± 0.46	*P* = 0.358
IL-12	1.09 ± 0.65	0.87 ± 0.57	*P* = 0.433	0.86 ± 0.63	0.55 ± 0.67	*P* = 0.672

### Reduction in HSCs in patients with AA

**Fig. 4:**
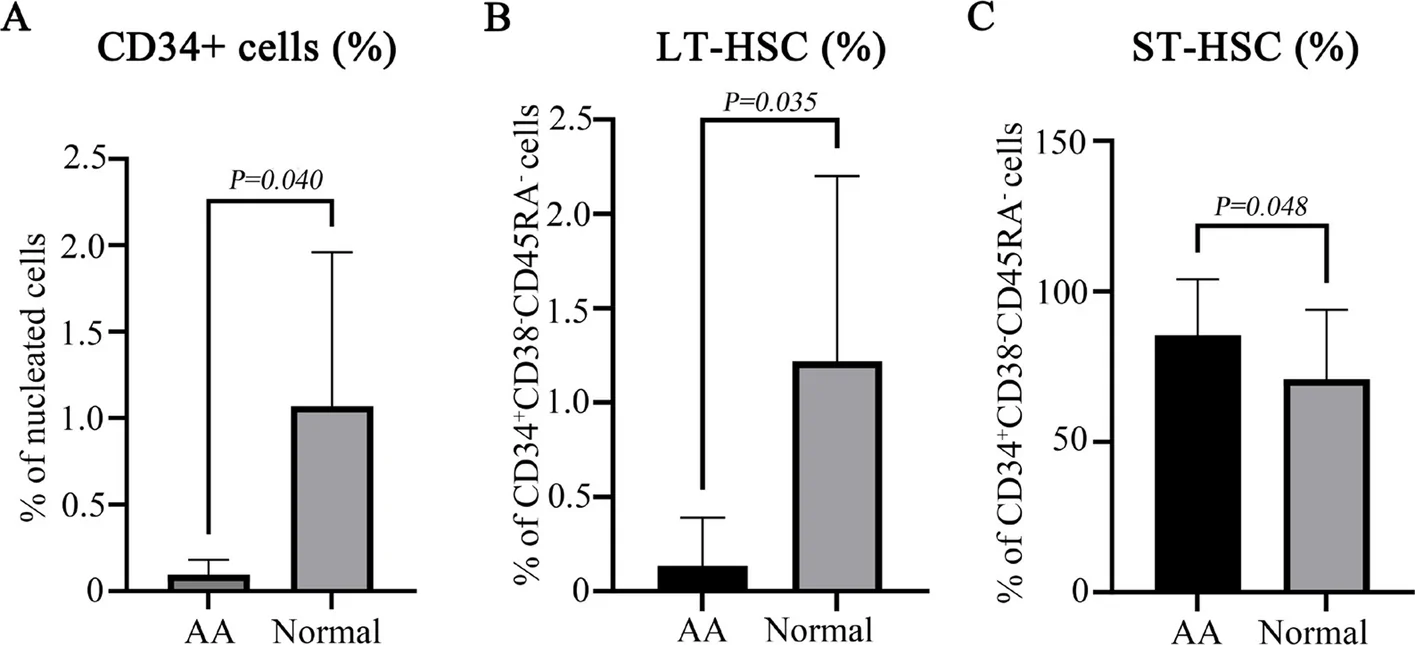
Bone marrow hematopoietic stem cell difference analysis: HSCs (**A**), LT-HSCs (**B**), and ST-HSCs (**C**). LT-HSCs and ST-HSCs in the bone marrow of patients with aplastic anemia, especially LT-HSCs, were significantly lower than those in the control group

Flow cytometry was used to analyze the quantity of bone marrow HSCs and their subsets (LT-HSCs and ST-HSCs) in patients with AA and normal controls. In comparison with the normal controls, patients with AA showed fewer HSCs (Fig. 4A). To understand the status of the subpopulations of HSCs in AA, we also evaluated the numbers of LT-HSCs- and ST-HSCs. Both were significantly lower in the bone marrow of patients with AA than in the normal controls (for LT-HSCs/lin^−^CD34^+^CD38^−^CD45RA: 0.13% *vs* 1.22%, *P* = 0.035; for ST-HSCs/lin^−^CD34^+^CD38^−^CD45RA^−^: 0.13% 85.44% *vs* 70.96% *P* = 0.048; Fig. 4B and C), which indicated that AA was associated with impairment of the bone marrow hematopoietic stem cell pool, particularly the critical LT-HSCs that sustain long-term hematopoiesis.

## Discussion

IP was initially believed to exist only in the eyes, testes, and brain, i.e., organs with blood-tissue barriers. Subsequent research expanded the concept of IP to many tissues, including hair follicles, intestinal mucosa, and lungs. IP actively guides and controls immune responses through various mechanisms to maintain the integrity of an immune-tolerant microenvironment. The bone marrow IP sites have been characterized using high-resolution in vivo imaging in animal experiments [3]. The basic cellular component of IP is Tregs, which are characterized by FoxP3 expression in the nucleus. Through the binding of membrane C-X-C chemokine receptor type 4 (CXCR4) with a large amount of the chemokine C-X-C motif chemokine ligand 12 (CXCL12) in the HSC niche, Tregs aggregate to the bone marrow near the endosteum surface of the trabecular bone to surround HSCs [13, 16], where the transplanted allogeneic HSCs could survive for a long time (30 days) without a myeloablative conditioning regimen before transplantation, which is the gold standard for IP identification. When the Tregs were removed from the bone marrow by deleting the CXCR4 receptor in mice, the transplanted allogeneic HSCs described in the above experiment could not survive in the osteoblast niche, and the expression levels of TNF-α and IFN-γ were enhanced while that of IL-10 decreased in the bone marrow under stress conditions. The HSCs located in an IP immune-tolerant microenvironment are LT-HSCs [3]. Subsequent studies have shown that LT-HSCs are over-mobilized under stress induction after Treg depletion [13, 17]. Therefore, in the bone marrow, IP not only provides an immune-tolerant microenvironment to protect LT-HSCs from various immune factors but also reduces the over-mobilization of LT-HSCs during stress and maintains the static state of LT-HSCs, which are stem cells that maintain lifelong hematopoiesis in the bone marrow.

The ability of Tregs to migrate to the bone marrow has been shown to be impaired in patients with AA, and the proportion of Tregs in the bone marrow detected by flow cytometry is also decreased in these patients [18, 19]. However, these studies did not focus on IP. Interestingly, a series of immune disorders of AA reported in the literature and in our previous studies showed surprising similarities to the excessive immune status caused by damaged bone marrow IP: The increased expression of TNF-α and IFN-γ in CD4 + and CD8 + T cells was similar to the cytokine profile in the IP damage assay (Treg deletion) [3, 20, 21], while the Th1/Th2 polarization found in a previous study of an AA animal model was highly consistent with the immune disorder caused by impaired IP function [8, 22]. However, previous research on Tregs in AA was limited to peripheral blood and bone marrow fluid [18, 23], without delving into the bone marrow microenvironment.

Only the distribution of Tregs, Th1/Th2 balance, and cytokine profiles detected in the bone marrow microenvironment can truly reflect the function of IP. However, none of the existing research has linked IP with immune abnormalities in AA, nor has there been any study on the local immune tolerance status of the bone marrow in AA [6]. Therefore, drawing on other experimental methods for the examination of IP function in local tissues [5–7], we tested the distribution of Tregs in the osteoblast niche area of the bone marrow by immunohistochemistry and detected cytokine levels in bone marrow fluid that had been diluted by blood as little as possible. The number of Tregs on the endosteal surface in patients with AA was significantly fewer than those in normal controls and patients with MDS, and this was accompanied by an increase in the Th1/Th2 ratio as well as the levels of TNF-α, IFN-γ, and IL-17 in the bone marrow; these findings were not observed in MDS, another disease characterized by bone marrow failure. These results suggest that IP is impaired in the bone marrow of children with AA.

Generally, LT-HSCs appropriately transition to activated ST-HSCs, which differentiate into various blood cells [24]. This transition increases during stress. Recent studies have shown that Tregs regulate the level of reactive oxygen species in LT-HSCs to prevent them from being over-mobilized and exhausted during oxidative stress [13, 15]. Our experiments showed significantly reduced LT-HSCs in AA, which could be attributed to the loss of the maintenance effect of IP on LT-HSCs. Various clinical studies have also suggested that the ability to maintain HSCs against stress is reduced in patients with AA. Some patients develop AA because of infection, exposure to radioactive substances, or certain drugs, and the severity of the disease is often aggravated during infection [1, 25]. In clinical practice, 60%−70% of children with non-severe AA (NSAA) showed evolution to severe AA (SAA) during continuous exposure to various environmental stresses [26, 27]. After the depletion of LT-HSCs, reserve HSCs for mobilization were inadequate, resulting in a corresponding reduction in ST-HSCs. Therefore, we observed a decrease in the number of ST-HSCs in the bone marrow of children with AA.

### Study limitations and future directions

First, as a clinical observational study, this study only provided correlative evidence between Treg dysfunction and LT-HSC depletion; the direct causal relationship requires further validation using animal models. Second, our exploration of the underlying mechanisms, such as adenosine signaling or specific Treg subsets, remains preliminary. Additionally, owing to ethical constraints, the control group consisted of patients with pediatric cancer without metastasis or infection, whose systemic inflammatory state may have confounded bone marrow immunophenotyping. Finally, variations in flow cytometry gating strategies could have influenced the absolute cell counts. These limitations highlight the need for cautious interpretation and validation in future studies.

## Conclusions

**Fig. 5:**
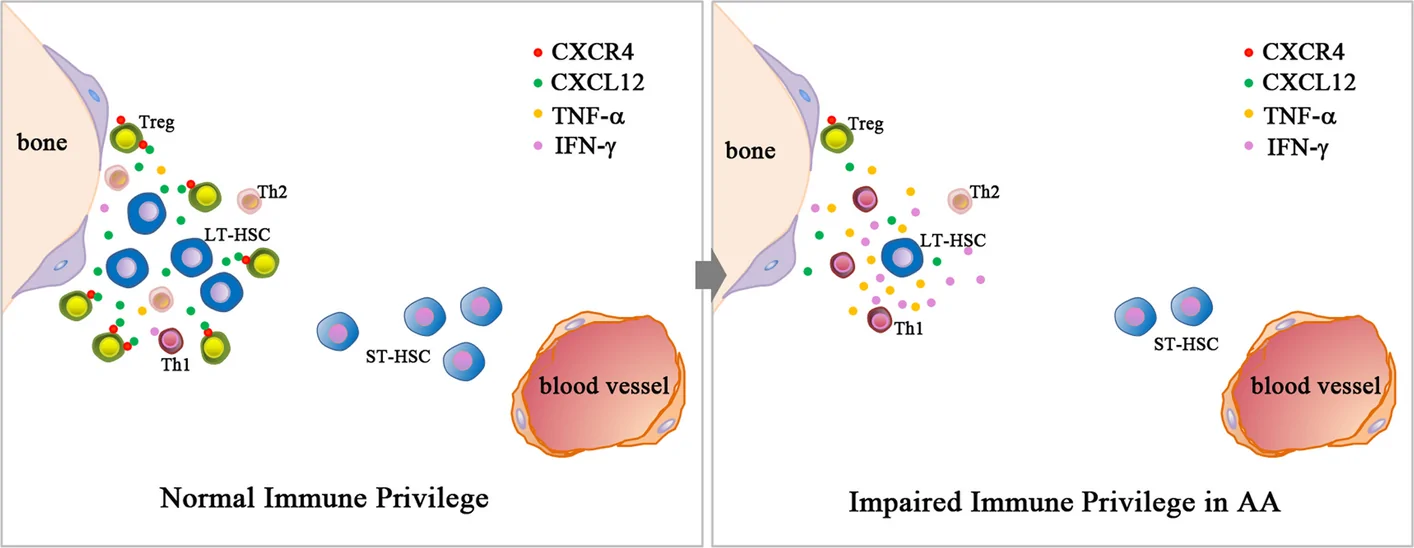
Schematic diagrams illustrating the impaired IP leading to HSC depletion in AA

Because HSCs are exhausted by the loss of IP protection, the alternative pathogenesis of AA proposed by us, wherein the specific depletion of HSCs is mediated by immune disorder in AA, can be reasonably explained as exhaustion of HSCs as a result of excessive mobilization under stress owing to the impaired immune tolerance microenvironment (Fig. 5). These findings can be expected to break through the existing bottleneck in studies of AA pathogenesis and warrant further study.

## Supplementary Information

Supplementary Material 1.

## Data Availability

The data that support the findings of this study are available from the corresponding author upon reasonable request.
