# Supplementary material for: The role of impaired bone marrow Tregs in hematopoietic stem cell depletion for pediatric aplastic anemia probably involves immune privilege
Source: Blood Res. 2026 May 26;61(1):29. doi: 10.1007/s44313-026-00141-6 (PMC13250033; doi:10.1007/s44313-026-00141-6)
Supplement: Supplementary file 1 — Supplementary Material 1. [file 44313_2026_141_MOESM1_ESM.docx]

**Supporting Information**

**The Role of Impaired Bone Marrow Tregs in Hematopoietic Stem Cell Depletion for Pediatric Aplastic Anemia Probably Involves Immune Privilege**

Can Huang ^1#^, Shanshan Li ^1#^, Jingwei Yang ^1^, Yangyang Jiao ^1^, Ting Zhang ^2^, Hui Jiang ^1^, Fanyi Zeng ^3,4*^, Shayi Jiang ^1,4*^.

^1^Department of Hematology and Oncology, Shanghai Children’s Hospital, School of Medicine, Shanghai Jiao Tong University, Shanghai, China

^2^Institute of Pediatric Infection, Immunity and Critical Care Medicine, Shanghai Children’s Hospital, School of Medicine, Shanghai Jiao Tong University, Shanghai, China

^3^Shanghai Institute of Medical Genetics, Shanghai Children's Hospital, & Department of Histo-Embryology, Genetics and Developmental Biology, School of Medicine, Shanghai Jiao Tong University, Shanghai, China

^4^NHC Key Laboratory of Medical Embryogenesis and Developmental Molecular Biology & Shanghai Key Laboratory of Embryo and Reproduction Engineering, Shanghai, China

^#^These authors contribute equally to the work

^*^Corresponding Author

Shayi Jiang, Email: jiangshayi@163.com; Fanyi Zeng, Email: [fzeng@vip.163.com](mailto:fzeng@vip.163.com)

**Keywords**: aplastic anemia, immune privilege, hematopoietic stem cells, immune microenvironment, regulatory T cells


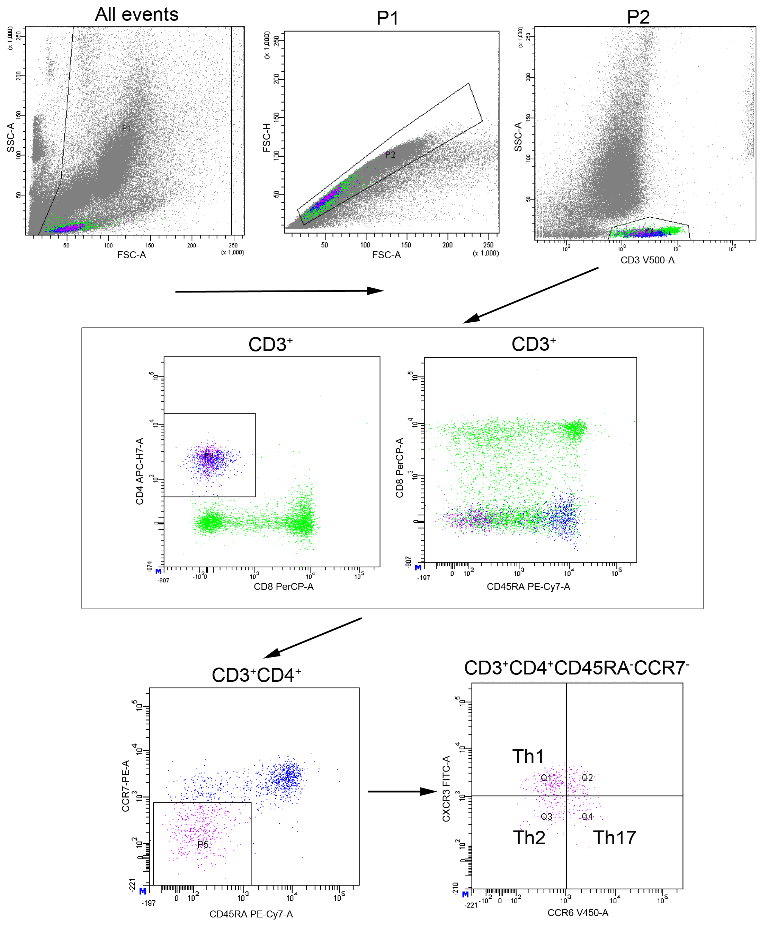


Supplementary Figure S1. Gating strategy used to identify Th1, Th2 and Th17 cells.


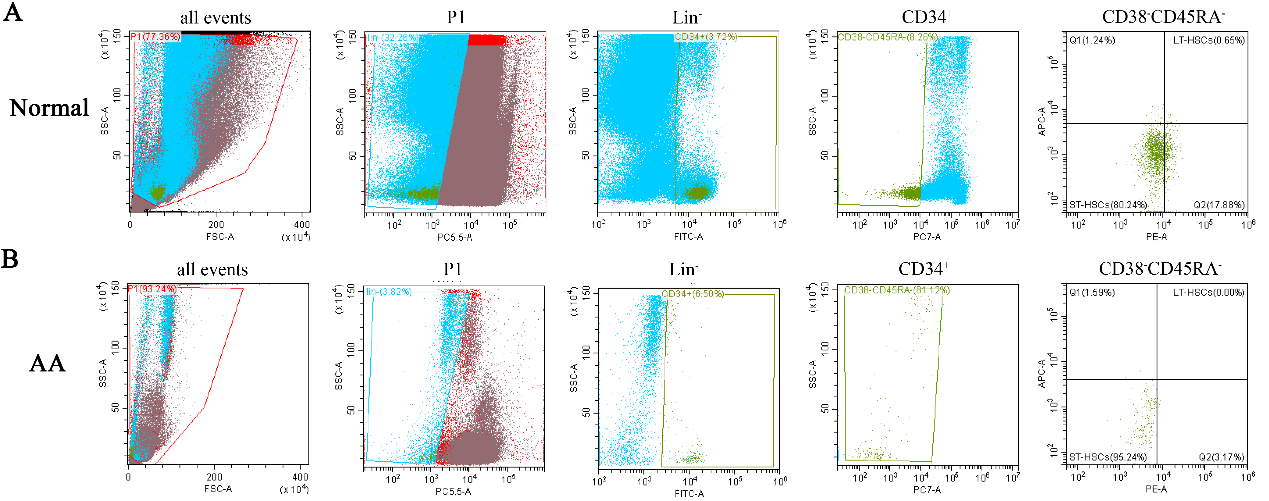


Supplementary Figure S2. Gating strategy used to identify LT-HSCs and ST-HSCs.
